# CELL SENESCENCE IS A FEATURE AND MODULATOR OF THE AGED INFLAMMATORY BRAIN CELL LANDSCAPE

**DOI:** 10.1093/geroni/igac059.781

**Published:** 2022-12-20

**Authors:** Marissa Schafer, Xu Zhang, Chase Carver, Vesselina Pearsall, Elizabeth Atkinson, Benjamin Clarkson, Ethan Grund, Nathan LeBrasseur

**Affiliations:** Mayo Clinic, Rochester, Minnesota, United States; Mayo Clinic, Rochester, Minnesota, United States; Mayo Clinic, Rochester, Minnesota, United States; Mayo Clinic, Rochester, Minnesota, United States; Mayo Clinic, Rochester, Minnesota, United States; Mayo Clinic, Rochester, Minnesota, United States; Mayo Clinic, Rochester, Minnesota, United States; Mayo Clinic, Rochester, Minnesota, United States

## Abstract

New strategies to preserve cognitive function could broadly benefit the aging population. Cellular senescence is a hypothesized mediator of inflammation-related tissue dysfunction in aging. Using single-cell RNA-sequencing, we discovered an age-related brain myeloid population exhibiting overlapping senescent and disease-associated activation signatures, including upregulation of chemoattractant factors. We confirmed senescent brain myeloid cells promote peripheral immune cell chemotaxis in vitro. Through mass cytometry, we demonstrate age- and sex-dependent increases in activated resident and infiltrating brain immune cells are reduced through systemic targeting of p16-positive senescent cells, which is associated with improvements in executive function and spatial learning tasks. Our high-dimensional results reveal dynamic remodeling of the age-dependent brain immune cell landscape and implicate senescent cell targeting as a strategy to counter inflammatory changes and cognitive decline.

